# Trends in Home Birth Information Seeking in the United States and United Kingdom During the COVID-19 Pandemic

**DOI:** 10.1001/jamanetworkopen.2021.10310

**Published:** 2021-05-17

**Authors:** Christina N. Schmidt, Laeesha N. Cornejo, Nicholas A. Rubashkin

**Affiliations:** 1 School of Medicine, University of California, San Francisco; 2 Department of Obstetrics and Gynecology, University of California, San Francisco

## Abstract

This cross-sectional study used online search data to assess changes in home birth information-seeking behaviors across the United States and United Kingdom during the COVID-19 pandemic.

## Introduction

COVID-19 has affected birthing practices on a global scale. Partners and doulas have been excluded from hospital birthing rooms,^1^ and patients have avoided hospital-based care.^2^ Simultaneously, home birth practitioners have reported increased interest in their services. In this analysis, we used online search data to assess changes in home birth information-seeking behaviors across the United States and United Kingdom during the COVID-19 pandemic.

## Methods

This cross-sectional study followed the Strengthening the Reporting of Observational Studies in Epidemiology (STROBE) reporting guideline.^3^ Google Trends is a public search volume database and has been used to understand changes in public interest in various health-related contexts.^4,5^ Because the tool is anonymized and publicly available, the University of California San Francisco did not require institutional review. Data in the tool are reported as weekly relative search volumes (RSVs), which reflect the query share of a search term relative to the total number of queries during a specified period, scaled from 0 to 100, with a higher score indicating a greater query share. Data were extracted for the following search terms, selected via the tool’s related queries database: *home birth*, *homebirth*, *birth at home*, *at home birth*, and *giving birth at home*. Queries from March 3, 2019, to November 1, 2020, in the United States and United Kingdom were extracted.

Interrupted time-series single-group analysis was performed to assess whether search frequency differed in the pre– and post–COVID-19 periods, using March 1, 2020, as the start of the COVID-19 period. We constructed segmented linear and nonlinear models to assess for differences in RSV trends between periods (2-tailed α = .05), while accounting for the slope of the underlying trend (Table).^6^ Models 1 and 2 were linear, while models 3 and 4 were nonlinear. Models 2 and 4 included terms accounting for the interaction between time and the pre– and post–COVID-19 indicator. Final model selection was based on mean squared error. Data were analyzed in R version 3.6.1 (R Project for Statistical Computing).

**Table.** Interrupted Time Series Model Selection

Model	United States		United Kingdom	
	Relative risk (95% CI)	*P* value	Relative risk (95% CI)	*P* value
1^a^	1.70 (1.51-1.93)	<.001	1.52 (1.32-1.75)	<.001
2^b^	3.39 (2.48-4.64)	<.001	1.53 (1.06-2.21)	.02
3^c^	1.82 (1.61-2.06)	<.001	1.49 (1.29-1.73)	<.001
4^d^	7.17 (0.77-67.12)	.08	4.21 (0.30-58.51)	.28

^a^ β_0_ + β_1_(COVID) + β_2_(Week).

^b^ Final model, selected based on mean squared error; β_0_ + β_1_(COVID) + β_2_(Week) + β_3_(Week × COVID).

^c^ β_0_ + β_1_(COVID) + β_2_(Week) + β_3_(Week^2^).

^d^ β_0_ + β_1_(COVID) + β_2_(Week) + β_3_(Week^2^) + β_4_ (Week × COVID) + β_5_(Week^2^ × COVID).

## Results

Mean (SD) RSV scores during the COVID-19 era were 53.5 (12.4) in the United States and 39.0 (10.9) in the United Kingdom, compared with pre–COVID-19 scores of 40.6 (14.3) and 31.2 (10.9), respectively. We found a 239% increase in home birth-related RSVs in the United States (relative risk [RR], 3.39; 95% CI, 2.48-2.64; *P* < .001) and a 53% increase in the United Kingdom (RR, 1.53; 95% CI, 1.06-2.21; *P* = .02) (Figure). Higher RSVs were noted in the earlier months of the pandemic, and 8 of 9 (89%) and 6 of 9 (67%) of the weeks with the highest volume of searches (ie, >90th percentile RSV score) in the United States and the United Kingdom, respectively, occurred between March and May 2020.

**Figure.**
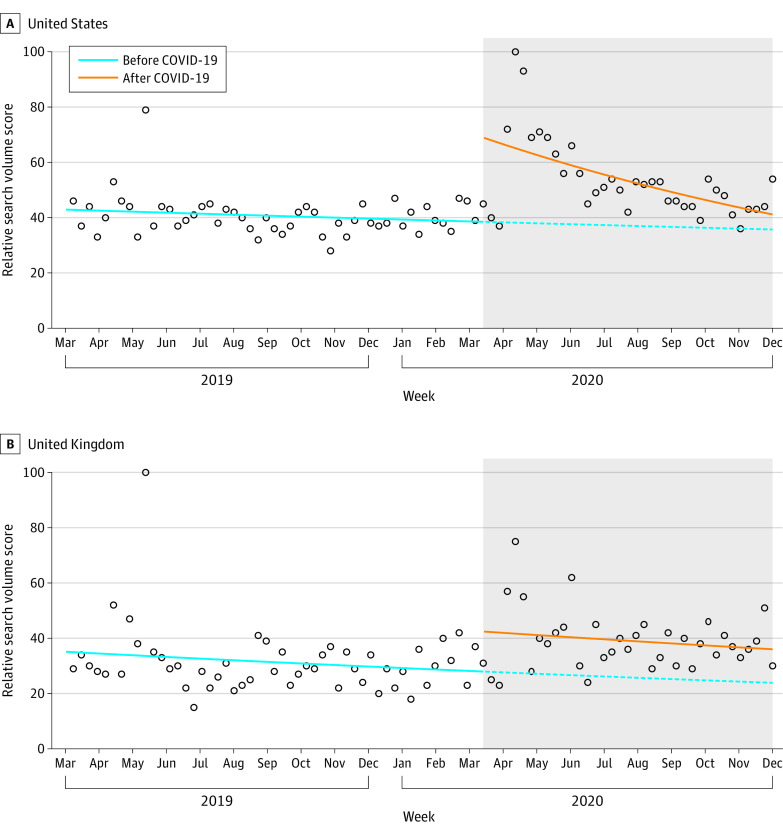
Relative Search Volume for Home Birth–Related Queries by Week in the United States and United Kingdom, March 2019 to November 2020 Circles represent weekly relative search volume scores; solid lines, actual RSV trend lines; dashed lines, estimated relative search volume trend lines in the absence of COVID-19; gray area, the COVID-19 period.

## Discussion

In this study, we identified increased public interest in home birth during the COVID-19 pandemic in the United States and the United Kingdom, most prominently during the early months of the outbreak. This increased information-seeking parallels media coverage and anecdotal reports from home birth practitioners, who have noted heightened demand for home birth services. While the largest spike in searches for home birth information occurred early in the pandemic, RSVs have persisted at levels greater than prepandemic trends. The association was more prominent in the United States than in the United Kingdom, which may reflect the United Kingdom’s higher baseline rate of home births and more integrated home birth system prior to the pandemic. These results have important implications for birth workers as they consider shifts in patient birthing preferences. Close collaboration between patients, home birth practitioners, and hospital-based practitioners is vital to promote resource sharing and optimize patient care.

As individuals increasingly rely on digital sources of health-related information, tools like the one used in this study are important for understanding information-seeking behaviors. In the context of an evolving pandemic, these tools allow for real-time analysis of rapidly changing trends at a population level. A limitation of this data set is that it only captures data from a single search engine. The population sample may be biased toward those with higher internet usage, including those who are literate and have internet access. In addition, search volume data may not necessarily correlate with utilization of services. Future research will be needed to understand the long-term association of increased public information seeking with the rates of home births.
